# Very-long-term outcomes of mechanical valves in mitral position focusing on valve-related complications

**DOI:** 10.1093/icvts/ivac146

**Published:** 2022-05-30

**Authors:** Gaku Uchino, Hirohisa Murakami, Nobuhiko Mukohara, Hiroshi Tanaka, Yoshikatsu Nomura, Shunsuke Miyahara, Motoharu Kawashima, Jun Fujisue, Shuto Tonoki

**Affiliations:** Department of Cardiovascular Surgery, Hyogo Brain and Heart Center at Himeji, Himeji, Japan

**Keywords:** Mechanical valve, Mitral valve replacement, Carbomedics, Paravalvular leak

## Abstract

**OBJECTIVES:**

This study aimed to examine very-long-term outcomes of a mechanical valve at the mitral position.

**METHODS:**

This study included all patients who underwent mitral valve replacement (MVR) using a mechanical valve including urgent operation at the Department of Cardiovascular Surgery, Hyogo Brain and Heart Center, Himeji, from January 1987 to December 2015.

**RESULTS:**

Five hundred and eighty-three patients (277 men [47.51%]; age, 61 [54–67] years) were included in this study. The implanted valve models were as follows: SJM, 221 (37.91%); ATS, 35 (6.00%); On-X, 68 (11.66%); and Carbomedics 194, (33.28%).The median clinical follow-up duration was 13.3 (7.4–19.6) years. The survival rates at 10, 15, 20 and 25 years were 81.42%, 69.27%, 56.34% and 45.03%, respectively. Thromboembolism was observed in 38 patients, and the linearized ratio for each event was 0.626%/patient-year [95% confidence interval (CI), 0.443–0.859%]. Intracranial haemorrhage and gastrointestinal bleeding were observed in 26 and 9 patients, and the linearized ratio for each event was 0.425%/patient-year (95% CI, 0.277–0.006%) and 0.145%/patient-year (95% CI, 0.067–0.276%), respectively. Major paravalvular leak was observed in 32 patients, and the linearized ratio was 0.532%/patient-year (95% CI, 0.364%–0.751%). The cumulative incidence rate of major paravalvular leak at 10, 15, 20 and 25 years was 3.7%, 5.6%, 6.4% and 10.4%, respectively. Multivariable Cox regression analysis revealed that repeated MVR and male gender were associated with major paravalvular leak.

**CONCLUSIONS:**

Male gender and repeated MVR were risk factors for paravalvular leak after mechanical MVR. Paravalvular leak could have occurred regardless of postoperative period even at 25 years after implantation. Lifelong clinical follow-up is considered necessary.

**Figure:**
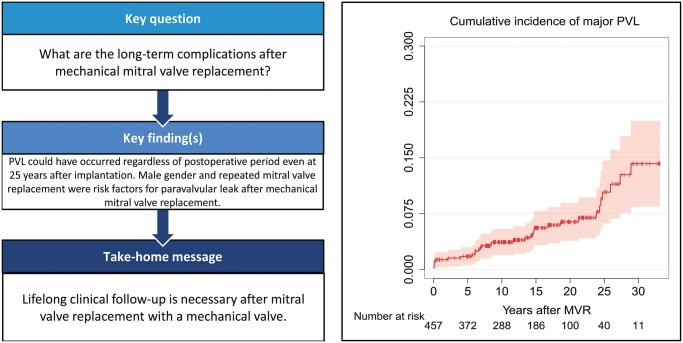

## INTRODUCTION

Although bioprosthetic mitral valve replacement (MVR) has been widely performed instead of mechanical MVR, structural valve deterioration could not be avoided. Especially for porcine valve, not only late valve failure but also early valve failure has been reported [1]. The mechanical valve, which guarantees long-term durability, remains a significant treatment option especially for young patients.

For middle-aged patients, the use of valve prosthesis is an important consideration [2].

Previous reports about a mechanical valve at the mitral position had a relatively short follow-up considering patient’s young age [3–5]. More than 20 years of life expectancy after MVR should be considered as an average lifetime would extend.

We analysed our experience of over 25 years of MVR using a mechanical valve focusing on long-term complications.

## MATERIALS AND METHODS

### Ethical statement

The Institutional Review Board of Hyogo Brain and Heart Center, Himeji, approved this observational study (approval number, R3-23; date, 10 August 2021), and additional patient consent was not required because the present study was retrospective and it used anonymous patient’s clinical data. In addition, we applied an opt-out method of our hospital website (www.hbhc.jp) to obtain consent on this study.

### Patients

This study included all patients who underwent MVR using a mechanical valve including urgent operation at the Department of Cardiovascular Surgery, Hyogo Brain and Heart Center, Himeji, from January 1987 to December 2015. Each patient’s characteristics (e.g., age, gender and body surface area) and mitral aetiology were collected from medical records and are shown in Table 1.

**Table 1:** Patient characteristics

Age, median (IQR)	61 (54–67)
Gender (male), *n* (%)	277 (47.51)
Body surface area (m^2^), median (IQR)	1.57 (1.37–1.70)
Atrial fibrillation, *n* (%)	272 (46.66)
Mitral aetiology, *n* (%)	
Mitral regurgitation	183 (31.39)
Functional mitral regurgitation	14 (2.40)
Mitral stenosis	91 (15.61)
Mitral stenosis and regurgitation	175 (30.02)
Infective endocarditis	67 (11.49)
Post-mitral valve plasty	27 (4.63)
Post-mitral valve replacement	33 (5.66)
Cause of implanted mitral valve failure	Structural valve deterioration: 19 Paravalvular leak: 4 Prosthetic valve endocarditis: 3 Pannus: 1 Unknown: 6

IQR: interquartile range.

## METHODS

At MVR surgery, the anterior leaflet and its subvalvular apparatus were resected, whereas posterior leaflets were preserved if possible. In the presence of a calcified annulus, small annulus, calcified or thickened leaflet, and infected leaflet, the leaflet and subvalvular tissue were resected. A mechanical valve was implanted in the intra-annular position in all cases. In all patients, operative findings (e.g., implanted valve brand, valve size, subvalvular apparatus preservation status and concomitant aortic valve replacement status) were recorded and are summarized in Table 2.

**Table 2:** Operative and postoperative findings

Implanted valve brand, *n* (%)	
SJM	221 (37.91)
ATS	35 (6.00)
On-X	68 (11.66)
Carbomedics	194 (33.28)
Unknown	65 (11.15)
Implanted valve size (mm), *n* (%)	
23	2 (0.34)
25	130 (22.30)
27	205 (35.16)
27–29	14 (2.40)
29	101 (17.32)
31	48 (8.23)
31–33	3 (0.51)
33	10 (1.72)
Posterior leaflet preservation, *n* (%)	435 (74.61)
Concomitant aortic valve replacement, *n* (%)	132 (22.64)
Concomitant tricuspid valve procedure, *n* (%)	68 (11.66)
Maze procedure, *n* (%)	46 (7.89)
Left appendage closure, *n* (%)	42 (7.2)
Concomitant coronary arterial bypass grafting, *n* (%)	33 (5.66)
In-hospital mortality, *n* (%)	27 (4.63)
Cause of in-hospital mortality	Low output syndrome: 8 Multi-organ failure: 5 Intestinal ischaemia: 2 Other causes: 12.

Postoperative anticoagulation therapy was conducted using warfarin. The international normalized ratio of prothrombin time (PT-INR) was controlled between 2.5 and 3.0 in reference to the prior study for Asian people [6–8]. Patients were followed up by blood examination and echocardiography in our hospital every 12 months.

Each patient’s short-term result was evaluated by in-hospital mortality.

The long-term result was assessed by survival and mitral valve-related complications [thromboembolism (stroke, organ embolism, and valve thrombosis), bleeding (intracranial and gastrointestinal), paravalvular leakage (PVL), pannus, prosthetic valve endocarditis and all-cause cardiac reoperation].

Major PVL was defined as PVL requiring reoperation due to haemolytic anaemia or heart failure.

### Statistical analysis

Continuous variables are represented as mean (standard deviation) or median (interquartile range), as appropriate. They were analysed using Student’s *t*-test or the Mann–Whitney *U*-test after confirmation that the data exhibited a normal distribution, using the Shapiro–Wilk test. Categorical variables were analysed using Fisher’s exact test. A *P*-value of <0.05 was considered statistically significant. The follow-up rate was calculated using Clark’s method [9]. The incidence of late death and events was expressed in a linearized form (percentage per patient-year) with 95% confidence interval (CI).

The correlation between 2 variables was analysed using Pearson’s correlation analysis. Survival curves were calculated using the Kaplan–Meier method.

Analysis of long-term result other than survival was performed using cumulative incidence function with death as competing event, and groups were compared with Gray’s test. A Fine–Gray competing risk regression model was used to estimate subdistribution hazard ratio (sHR) along with 95% CI. For >3 groups, pairwise *post hoc* analyses were performed using the Bonferroni test to correct for multiple comparisons. Factors associated to major PVL were analysed using the Cox regression analysis. Variables of age, male gender, infective endocarditis, repeated MVR and implant technique (posterior leaflet preservation), which were considered relevant to PVL from prior study [10–14], were selected. Among these, variables that reached *P* < 0.1 on univariable Cox regression analysis (male gender, repeated MVR and posterior leaflet preservation) were introduced to multivariable analysis.

All analyses were conducted using Stata version 15.0 (StataCorp, College Station, TX, USA) and EZR software, version 1.54.

## RESULTS

This study included 583 patients (277 men [47.51%]; age, 61 [54–67] years). The median clinical follow-up duration was 13.3 (7.4–19.6) years. Preoperative patient characteristics are summarized in Table 1. Operative data are summarized in Table 2.

### Short-term results

In-hospital death was observed in 27 (4.63%) patients. Their cause of death is summarized in Table 2.

### Long-term results

The follow-up rate was 64.5%. Late death was observed in 189 patients, and the linearized ratio for late death was 3.047%/patient-year (95% CI, 2.628–3.514%).

**Figure 1:**
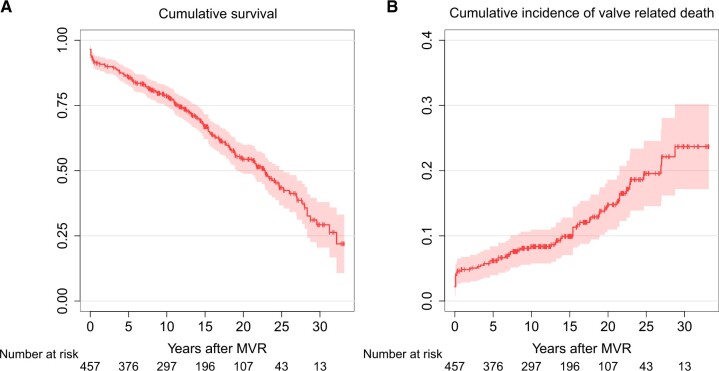
(**A** and **B**) Rates of survival and cumulative incidence of valve-related death.

The survival rates at 10, 15, 20 and 25 years were 81.4%, 69.3%, 56.3% and 45.0%, respectively, as shown in Fig. 1A. The survival rate according to operative indication is summarized in Supplementary Material, Fig. S1.

Valve-related death was observed in 64 patients, and the linearized ratio for valve-related death was 1.032%/patient-year (95% CI, 0.795–1.318%).

The cumulative incidence rate of valve-related death at 10, 15, 20 and 25 years was 8.4%, 9.9%, 14.8% and 19.5%, respectively, as shown in Fig. 1B.

### Thromboembolism (stroke, organ embolism and valve thrombosis)

Thromboembolism was observed in 38 patients, and the linearized ratio was 0.626%/patient-year(95% CI, 0.443–0.859%). Details about thromboembolism are summarized in Table 3. For 38 patients with thromboembolism, 27 (71.1%) had developed atrial fibrillation (AF) at the time of thromboembolism and had not undergone left atrial appendage (LAA) closure. The causes of thromboembolism in the remaining 11 patients were insufficient warfarin control (PT-INR <1.8) in 6 patients, amyloidosis-associated minor stroke in 1 patient, patent foramen ovale in 1 patient and unknown in 3 patients.

**Table 3:** Details of valve-related complication

1) Thromboembolism	38
Thromboembolism-related death, *n* (%)	16 (42.1)
Recurrent thromboembolism, *n* (%)	5 (13.2)
PT-INR at thromboembolism, median (IQR)	1.61(1.41–2.06)
Age at thromboembolism, median (IQR)	72.0 (62.9–78.7)
Time until thromboembolism after surgery, median (IQR)	9.7 (4.6–14.6)
Breakdown of thromboembolism	
Major stroke (NIHSS ≥5)	22 (Including 4 haemorrhagic infarction)
The sites of embolization for major stroke	Bilateral ICA: 1, left MCA: 3, left PCA: 1, right MCA: 11, BA: 1, unknown: 5
Minor stroke (NIHSS <5)	11
Limb embolism	3
Valve thrombosis	2
Left atrial thrombus	1
2) Intracranial haemorrhage	27
Intracranial haemorrhage-related death, *n* (%)	15 (55.6)
Recurrent Intracranial Haemorrhage, *n* (%)	2 (7.4)
PT-INR at Intracranial Haemorrhage, median (IQR)	2.93 (2.35–5.19)
Age at Intracranial Haemorrhage, median (IQR)	70.8 (66.5–79.4)
Time until intracranial Haemorrhage after surgery, median (IQR)	9.6 (6.1–13.5)
Breakdown of intracranial haemorrhage	
Subdural haematoma	9
Intracerebellar haemorrhage	7
Chronic subdural haematoma	4
Traumatic subarachnoid haemorrhage	4
Subarachnoid haemorrhage	2
Acute epidural haematoma	1
3) Major PVL	32
Reoperation for major PVL	27 (repeat MVR: 20, PVL closure: 7)
Recurrent PVL after reoperation for major PVL	5
Major PVL-related death	4
Time until major PVL after surgery, median (IQR)	10.1 (4.8–19.9) years
4) All-cause cardiac reoperation	39
Breakdown of cardiac reoperation	
Mitral PVL	27
Aortic valve aetiology	7
Mitral valve thrombosis	1
Mitral PVE	3
Left atrial thrombus	1

BA: basilar artery; ICA: internal carotid artery; IQR: interquartile range; MCA: middle cerebral artery, MVR: mitral valve replacement; NIHSS: National Institutes of Health Stroke Scale; PCA: posterior cerebral artery; PT-INR: the international normalized ratio of prothrombin time; PVE: prosthetic valve endocarditis; PVL: paravalvular leak.

**Figure 2:**
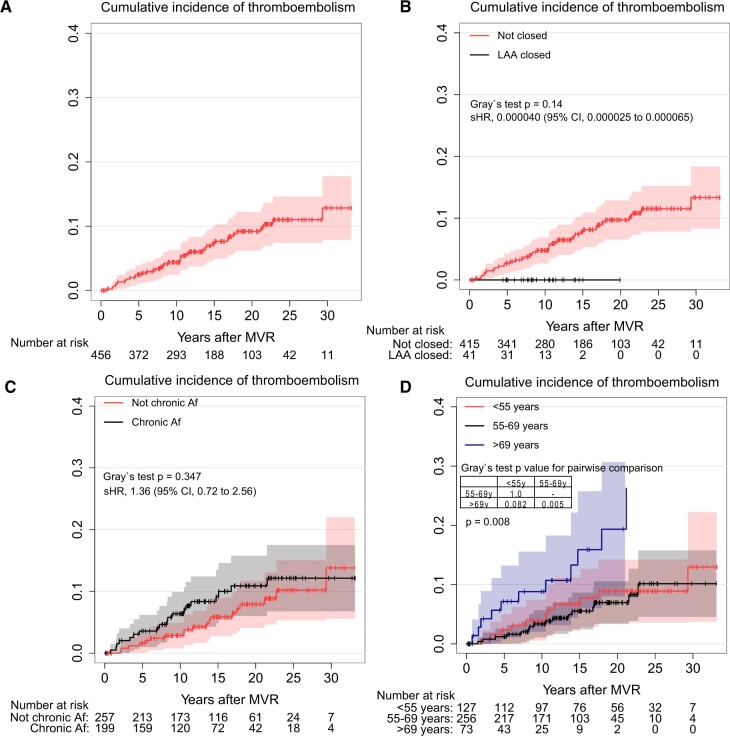
(**A**) Cumulative incidence of thromboembolism. (**B**) Cumulative incidence of thromboembolism with and without left atrial appendage closure. (**C**) Cumulative incidence of thromboembolism with and without chronic atrial fibrillation. (**D**) Cumulative incidence of thromboembolism stratified by age group.

The cumulative incidence rate of thromboembolism at 10, 15, 20 and 25 years was 4.4%, 7.3%, 9.2% and 11.0%, respectively, as shown in Fig. 2A. No LAA closure was noted, and preoperative chronic persistent AF tended to develop thromboembolism, although it was not statistically significant (sHR 0.000040, 95% CI 0.000025–0.000065, Gray’s test *P* = 0.14 as shown in Fig. 2B, sHR 1.36, 95% CI 0.72–2.56, Gray’s test *P* = 0.347 as shown in Fig. 2C).

The cumulative incidence rate of thromboembolism stratified by age groups (<55, 55–69 and >69 years) is shown in Fig. 2D. *Posthoc* analyses revealed that thromboembolism occurred more frequently for patients over 70 years (*P* = 0.008).

### Bleeding (intracranial haemorrhage/gastrointestinal bleeding)

Intracranial haemorrhage (ICH) and gastrointestinal bleeding were observed in 27 and 9 patients, respectively. The linearized ratios for each event were 0.440%/patient-year (95% CI, 0.291–0.006%) and 0.145%/patient-year (95% CI, 0.067–276%), respectively. Details about bleeding events are summarized in Table 3. Time until ICH (years) was moderately related to patient’s age (at MVR) (*r* = −0.415, *P* = 0.032).

**Figure 3:**
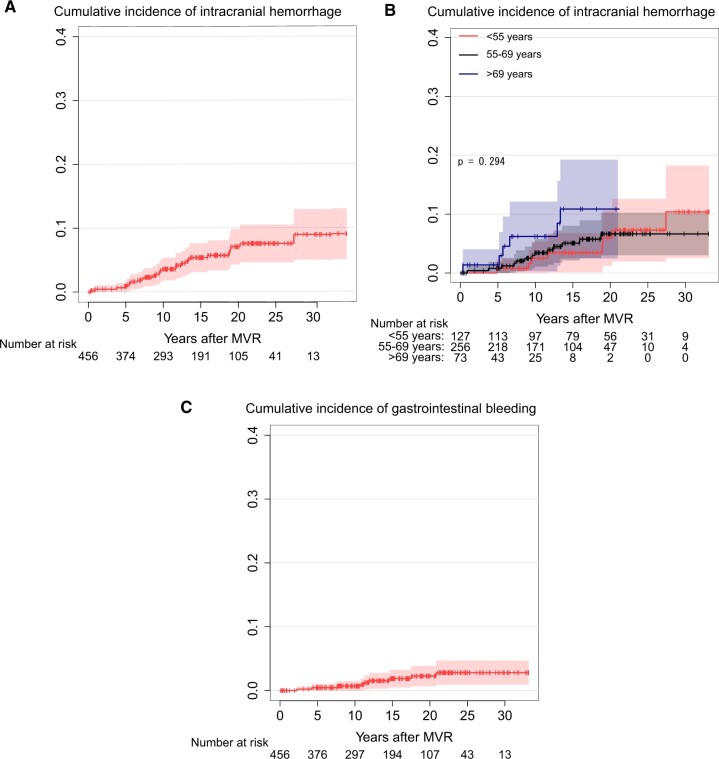
(**A**) Cumulative incidence of intracranial haemorrhage. (**B**) Cumulative incidence of intracranial Haemorrhage stratified by age group. (**C**) Cumulative incidence of gastrointestinal bleeding.

The cumulative incidence rate of ICH at 10, 15, 20 and 25 years was 3.6%, 5.4%, 7.1% and 7.6%, respectively, as shown in Fig. 3A. The cumulative incidence rate of ICH stratified by age groups (<55, 55–69, and >69 years) is shown in Fig. 3B. The difference between groups was not statistically significant (*P* = 0.294).

The cumulative incidence rate of gastrointestinal bleeding at 10, 15, 20 and 25 years was 0.7%, 1.9%, 2.3% and 2.8%, respectively, as shown in Fig. 3C.

### Major paravalvular leakage

Major PVL was observed in 32 patients, and the linearized ratio was 0.532%/patient-year (95% CI , 0.364–0.751%). Details about major PVL are summarized in Table 3.

**Figure 4:**
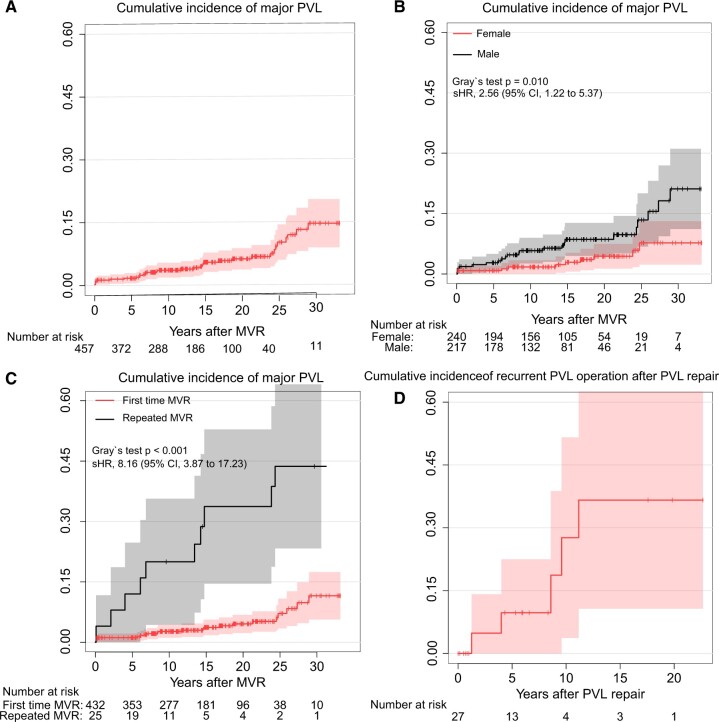
(**A**) Cumulative incidence of major paravalvular leak. (**B**) Cumulative incidence of major paravalvular leak in the male and female genders. (**C**) Cumulative incidence of major paravalvular leak in first-time mitral valve replacement and repeated mitral valve replacement. (**D**) Cumulative incidence of recurrent paravalvular leak operation after paravalvular leak repair.

The site of mitral PVL is shown in Supplementary Material, Fig. S2 using a clockwise format as viewed from the left atrium, according to a previous report [13]. Major PVL was mainly located at the 2 and 6 o’clock positions. Focusing on major late PVL (occurred after 100 months), it was mainly located at the 6 o’clock position. The cumulative incidence rate of major PVL at 10, 15, 20 and 25 years was 3.7%, 5.6%, 6.4% and 10.4%, respectively, as shown in Fig. 4A. The Gray’s test revealed that major PVL occurred more frequently in the male gender (sHR 2.56, 95% CI 1.22–5.37, *P* = 0.01) and repeated MVR (sHR 8.16, 95% CI 3.87–17.23, *P* < 0.001) (Fig. 4B and C), although the difference was not significant in the presence or absence of IE (sHR 0.43, 95% CI 0.11–1.74, *P* = 0.235), posterior leaflet preservation (sHR 0.60, 95% CI 0.29–1.26, *P* = 0.198) and depressed EF (sHR 1.02, 95% CI 0.35–2.95, *P* = 0.92) (Supplementary Material, Figs. S3–S5). Multivariable Cox regression analysis revealed that repeated MVR and male gender were associated with major PVL, as shown in Table 4.

**Table 4:** Univariable and multivariable Cox regression analyses to identify factors associated to major paravalvular leak

Variable	Univariate		Multivariate	
	HR (95% CI)	*P*-Value	HR (95% CI)	*P*-Value
Age	1.016 (0.975–1.058)	0.451		
Male	2.750 (1.266–5.976)	0.011	3.393 (1.523–7.559)	0.003
Infective endocarditis	0.466 (0.111–1.957)	0.297		
Repeated MVR	9.272 (4.334–19.839)	<0.001	13.401 (5.045–35.601)	<0.001
Posterior leaflet preservation	0.530 (0.249–1.126)	0.098	1.449 (0.550–3.819)	0.453

CI: confidence interval; HR: Hazard ratio; MVR: mitral valve replacement.

For 27 patients who underwent PVL reoperation, the cumulative incidence rate of recurrent PVL operation at 5 and 10 years are 9.7% and 27.6%, respectively, as shown in Fig. 4D.

Among those 27 patients, 5 patients underwent third time mitral valve surgery due to recurrent PVL after 8.6 (4–9.6) years after PVL reoperation.

### Pannus

Pannus formation and its related reoperation were not observed in the study cohort.

An increase in the transmitral mean pressure gradient (TMPG) (>5 mmHg) without PVL was observed in 71 patients. The median TMPG during follow-up was 4.1 (3.3–5.5) mmHg.

### Prosthetic valve endocarditis

Prosthetic valve endocarditis was observed in 6 patients, and the linearized ratio was 0.100%/patient-year (95% CI, 0.036–0.218%). For these 6 patients, no patient developed PVL.

### All-cause cardiac reoperation

Cardiac reoperation was observed in 39 patients, and the linearized ratio was 0.651%/patient-year (95% CI, 0.463–0.890%). Details about cardiac reoperation are summarized in Table 3. The cumulative incidence rate of cardiac reoperation at 10, 15, 20 and 25 years was 4.4%, 6.3%, 7.5% and 12.5%, respectively.

## DISCUSSION

### Thromboembolism

The target level of PT-INR in this cohorts was regulated between 2.5 and 3.0, which was consistent with prior Asian study [6–8]. Although it was lower than the European guideline [15], Asian people were considered vulnerable to warfarin and bleeding [8].

In the present study, the median PT-INR at thromboembolism was lower than the therapeutic target level, and this even was considered to be associated with poor warfarin administration.

The incidence of thromboembolism was consistent compared with previous reports(1.1%/patient-year) [16]. Chronic AF and no maze procedure were reported to be risk factors for late stroke after mechanical MVR [17]. These data suggested that late stroke after mechanical MVR was due to left atrial thrombus. In the present study, no LAA closure was noted, and chronic persistent AF tended to develop thromboembolism, although it was not statistically significant possibly because of the small sample number.

### Intracranial haemorrhage

Regarding ICH, most cases (18/27 patients) were subdural/epidural haematoma and traumatic SAH, which were due to contusion. Preventing head trauma and proper warfarin administration were considered to be significant. Patient’s age at surgery and time until ICH (years) were inversely correlated. It maybe that older patients tend to be at higher risk of all and head trauma.

### Pannus

Pannus formation was reported to occur more frequently in the aortic position and in female patients [18, 19]. The incidence of pannus formation after mechanical MVR was reportedly below 1% [16], which is consistent with the present study (0%).

Pannus formation at prosthetic mitral valve maybe underdiagnosed through transthoracic echocardiography. Chang *et al.* [20] reported that computed tomography was a more sensitive tool for diagnosing mitral pannus formation, and nearly 30% of their cohorts developed mitral pannus. In our cohorts, patients with an increased TMPG during follow-up may have developed mitral pannus unconsciously.

### Paravalvular leakage

The most notable finding of the present study is PVL.

Major PVL had occurred even after 25 years from implantation. It is suggested that long-term annular stress would cause valve dehiscence even at 25 years after implantation. Major late PVL was mainly located at the 6 o’clock position. It is suggested that the posterior mitral annulus, which does not have intervalvular fibrous body, would be deteriorated for a long time and result in tissue cutting and valve dehiscence.

Mitral PVL was reported to be relatively higher than aortic PVL [4, 16, 21].

This tendency is similar to the incidence of structural valve deterioration of the bioprosthetic valve [16, 21, 22]. Systolic high pressure gradient between the left atrium and ventricle would contribute to a higher risk of mitral PVL occurrence. Moreover, mitral annular motion during cardiac cycle may promote prosthetic valve dehiscence.

The Cox regression analysis revealed that repeated MVR and male gender were associated with PVL, although most (29 of 33) repeated MVR were underwent due to causes other than PVL. It is considered that prosthetic valve explants would injure the mitral annulus or the mitral annulus covered by granulation tissue would affect accurate suture during valve implantation. Moreover, men were more likely to develop PVL, which might be due to more physical activity [10]. We speculated that female patients were more likely to make pannus, which adhered prosthetic valve and mitral annulus, although it was haemodynamically insignificant and prevented prosthetic valve detachment as pointed out by Chang *et al.* [20].

Although previous reports proposed the relation of the valve type as the causes of PVL [10, 14], the present study could not prove the relation of such factors. Theoretically, the annular stress and healing process after MVR were considered to be different owing to the valve type. The valve closing behaviour and associated increase in the left ventricular pressure were different by valve types [23] and may influence annular stress. The increase in the sample size may prove involvement of such factors.

### Limitations

Our study has limitations. First, the study is a retrospective study from a single institution. In addition, sample size is small considering study period. Changes of intraoperative and perioperative strategy through long study period may make the study cohorts heterogeneous. Moreover, old clinical data were partly not complete and resulted to a relatively low follow-up rate. Definition of complication after surgery was partly different from Valve Academic Research Consortium [24, 25] definition. As a clinical follow-up, transoesophageal echocardiography was not performed routinely, and there would be potential underdiagnosis of PVL or pannus.

## CONCLUSION

Male gender and repeated MVR were risk factors for PVL after mechanical MVR.

PVL could have occurred regardless of postoperative period even at 25 years after implantation. Lifelong clinical follow-up is considered necessary.

## SUPPLEMENTARY MATERIAL

Supplementary material is available at *ICVTS* online.

**Conflict of interest:** none declared.

### Data Availability Statement

The data associated with the study are not publicly available but are available from the corresponding author on reasonable request.

### Author contributions

**Gaku Uchino:** Conceptualization; Data curation; Formal analysis; Investigation; Writing – original draft. **Hirohisa Murakami:** Conceptualization; Supervision. **Nobuhiko Mukohara:** Supervision. **Hiroshi Tanaka:** Conceptualization; Supervision. **Yoshikatsu Nomura:** Conceptualization; Supervision. **Shunsuke Miyahara:** Conceptualization; Supervision. **Motoharu Kawashima:** Conceptualization. **Jun Fujisue:** Data curation. **Shuto Tonoki:** Data curation.

### Reviewer information

Interactive CardioVascular and Thoracic Surgery thanks Donald D. Glower, Laura S. Fong and the other, anonymous reviewer(s) for their contribution to the peer review process of this article.

## Supplementary Material

ivac146_Supplementary_Data

## ABBREVIATIONS

AF: Atrial fibrillation
CI: Confidence interval
ICH: Intracranial haemorrhage
LAA: Left atrial appendage
MVR: Mitral valve replacement
PT-INR: The international normalized ratio of prothrombin time
PVL: Paravalvular leakage
sHR: Subdistribution hazard ratio
TMPG: Transmitral mean pressure gradient
