# Supplementary material for: Very-long-term outcomes of mechanical valves in mitral position focusing on valve-related complications
Source: Interact Cardiovasc Thorac Surg. 2022 May 30;35(2):ivac146. doi: 10.1093/icvts/ivac146 (PMC9297525; doi:10.1093/icvts/ivac146)
Supplement: ivac146_Supplementary_Data [file ivac146_supplementary_data.zip › figS1.pdf]

Cumulative survival according to operative indication

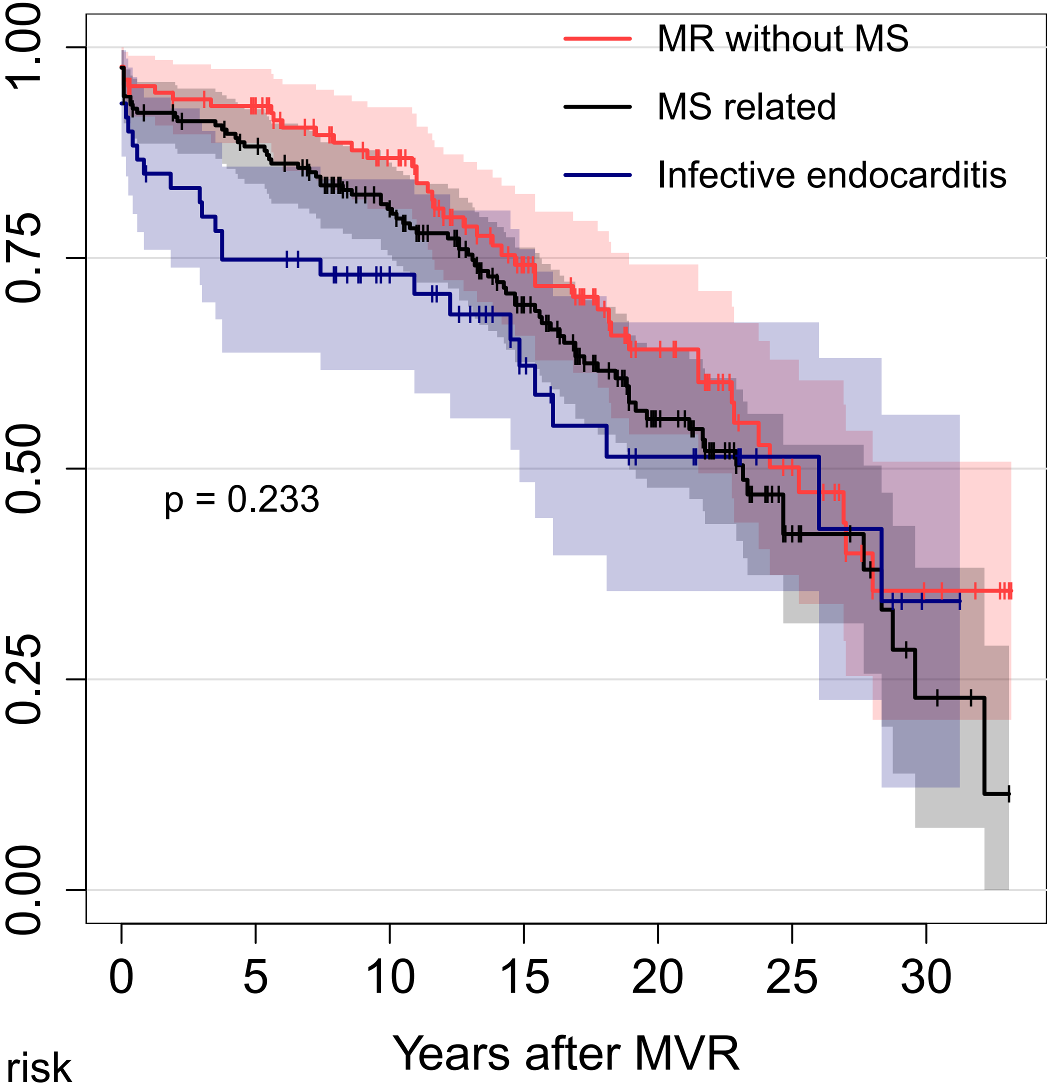

Number at risk							
MR without MS:	130	114	91	62	37	18	6
MS related:	206	175	143	100	51	16	4
IE:	60	44	33	19	11	6	1
