# Supplementary figures and images for: Very-long-term outcomes of mechanical valves in mitral position focusing on valve-related complications
Source: Interact Cardiovasc Thorac Surg. 2022 May 30;35(2):ivac146. doi: 10.1093/icvts/ivac146 (PMC9297525; doi:10.1093/icvts/ivac146)

# Cumulative incidence of major PVL

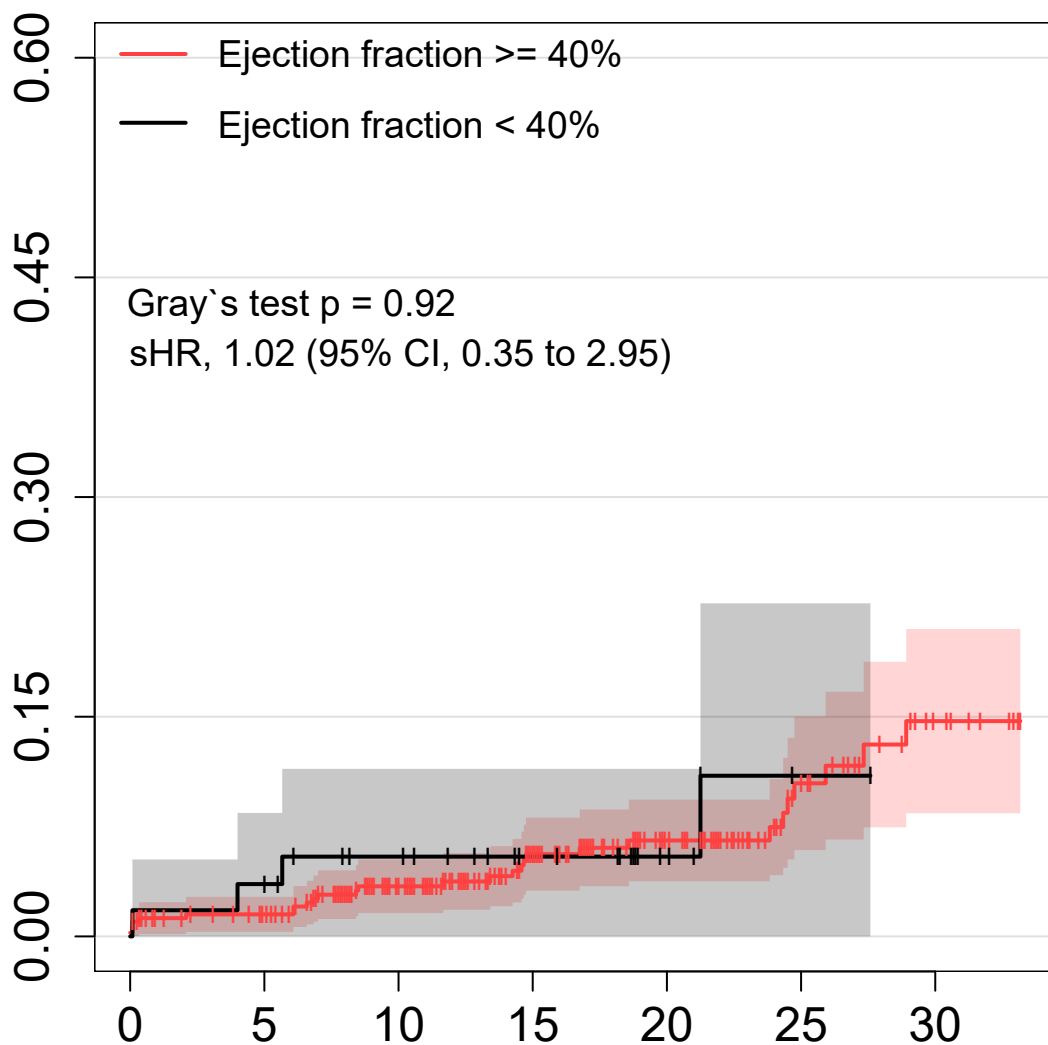

Number at risk

|                  |     |     |     |     |    |    |    |
|------------------|-----|-----|-----|-----|----|----|----|
| EF $\geq 40\%$ : | 401 | 330 | 253 | 165 | 89 | 38 | 11 |
| EF $< 40\%$ :    | 56  | 42  | 35  | 21  | 11 | 2  | 0  |

Supplement: ivac146_Supplementary_Data [file ivac146_supplementary_data.zip › figS5_2.pdf]

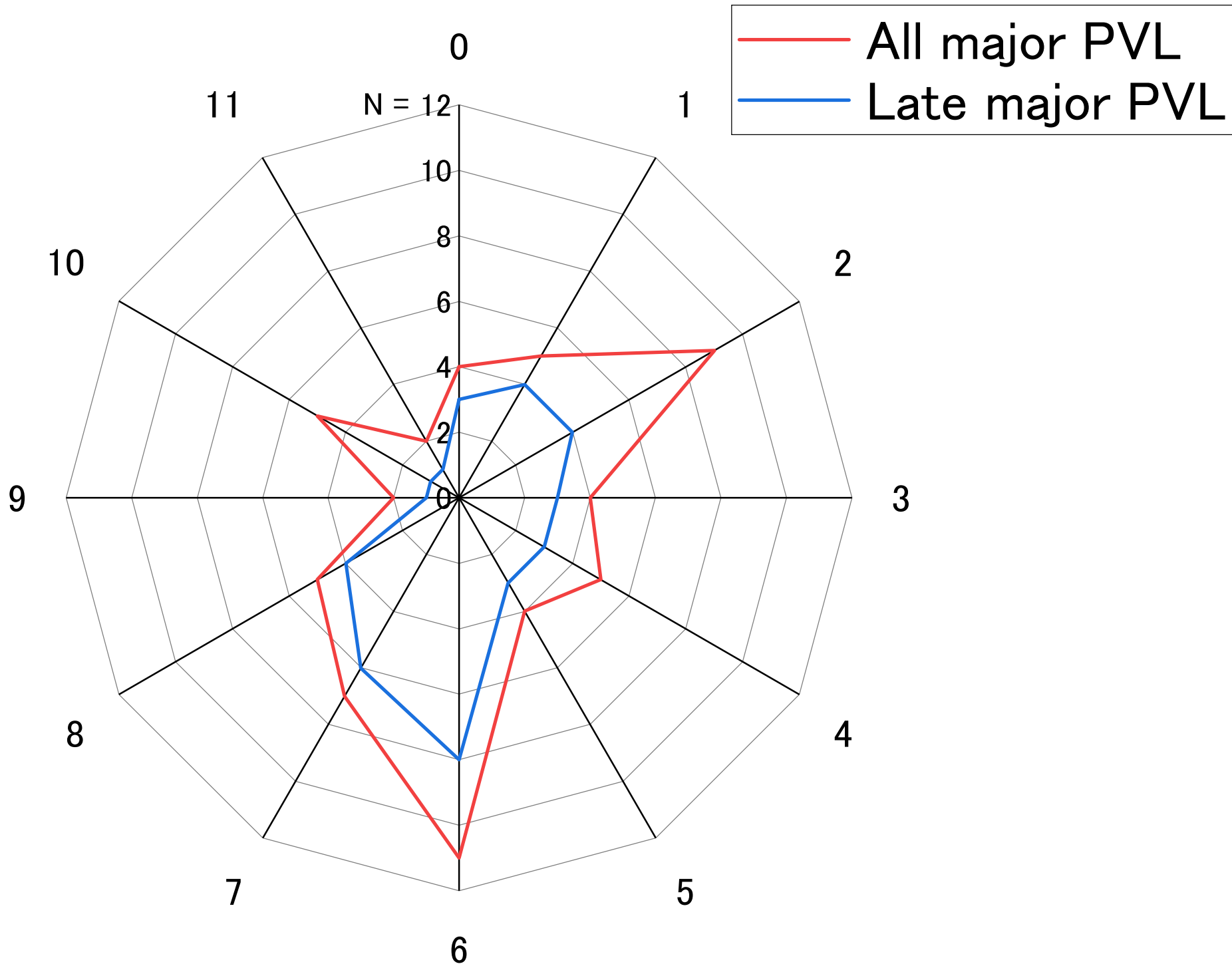

Supplement: ivac146_Supplementary_Data [file ivac146_supplementary_data.zip › figS2.pdf]

# Cumulative incidence of major PVL

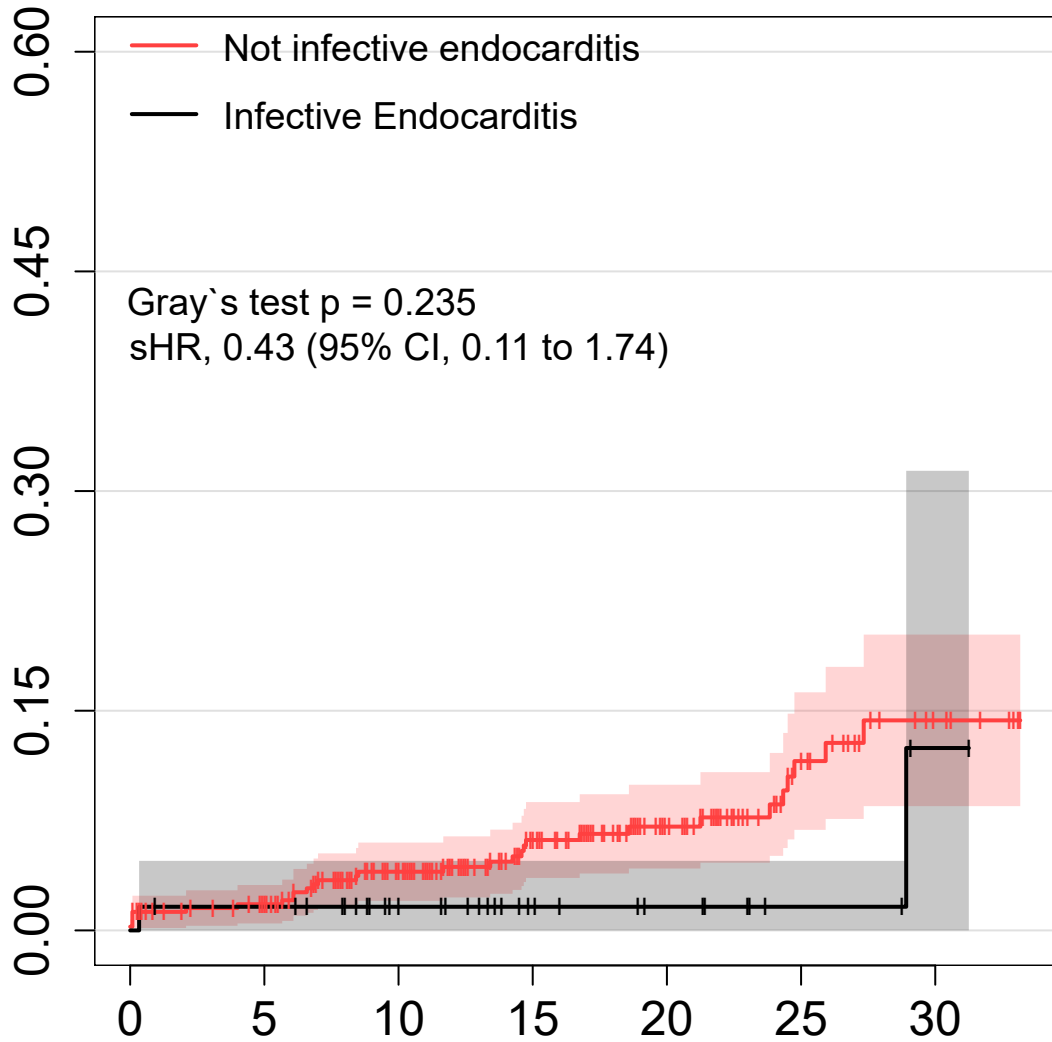

Number at risk

Years after MVR

Not IE: 395  
IE: 62

327  
45

254  
34

167  
19

89  
11

34  
6

10  
1

Supplement: ivac146_Supplementary_Data [file ivac146_supplementary_data.zip › figS3_2.pdf]

# Cumulative incidence of major PVL

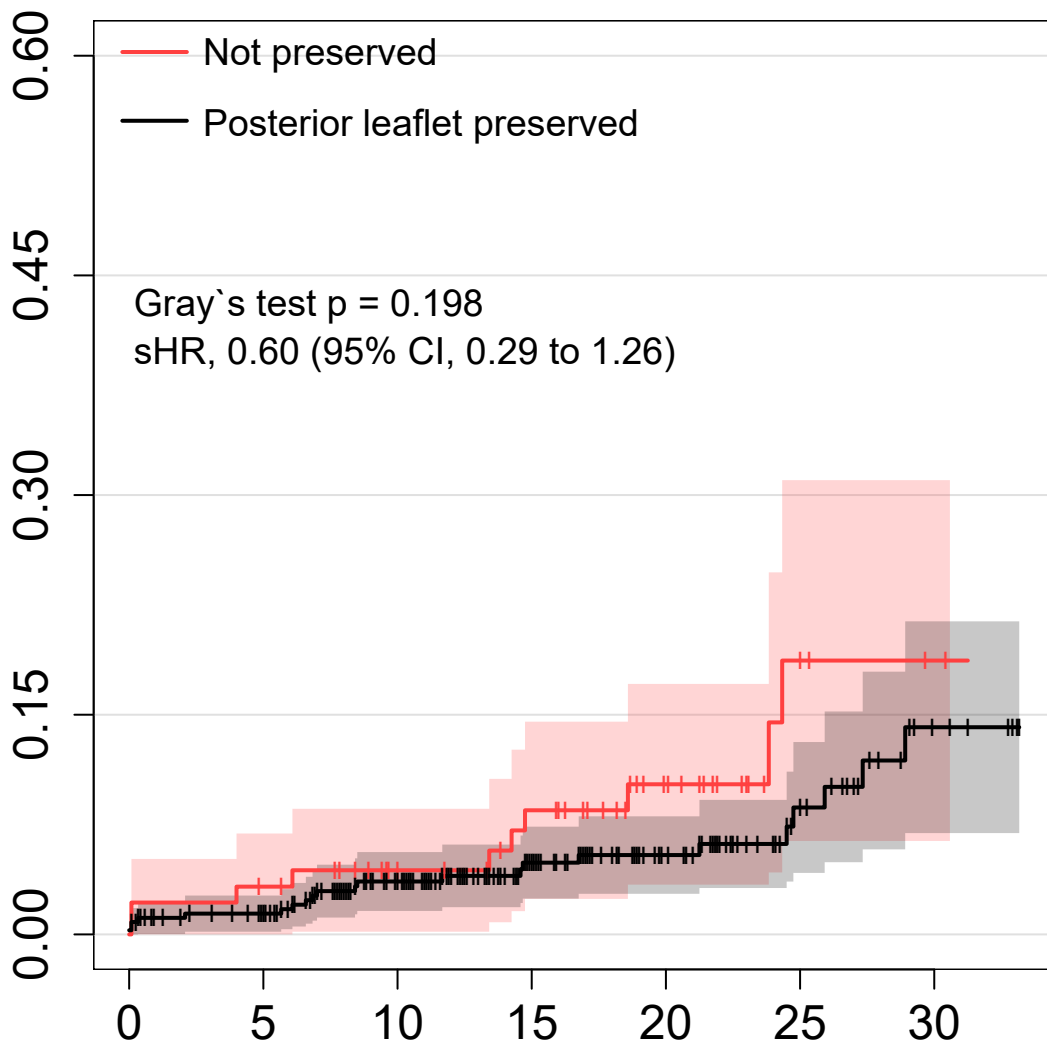

| Years after MVR |     |     |     |     |    |    |   |
|-----------------|-----|-----|-----|-----|----|----|---|
| Number at risk  |     |     |     |     |    |    |   |
| Not preserved:  | 92  | 70  | 52  | 40  | 21 | 7  | 2 |
| PL preserved:   | 355 | 292 | 226 | 138 | 73 | 28 | 6 |

Supplement: ivac146_Supplementary_Data [file ivac146_supplementary_data.zip › figS4_2.pdf]
